# Association très rare: luxation de l’épaule et disjonction acromio-claviculaire

**DOI:** 10.11604/pamj.2014.18.244.4892

**Published:** 2014-07-25

**Authors:** Younes Ouchrif, Issam Elouakili

**Affiliations:** 1 Service de Chirurgie Orthopédique, CHU de Rabat, Maroc

**Keywords:** Luxation, épaule, disjonction acromio-claviculaire, dislocation, shoulder, acromioclavicular disjunction

## Image en medicine

L'association luxation gléno humérale et disjonction acromio claviculaire est exceptionnelle. Rares cas sont décrits dans la littérature. Elles sont dues à une chute sur le moignon de l’épaule entrainant des lésions des moyens d'instabilités gléno humérales et aussi des lésions des ligaments conoïde et trapézoïde de l'articulation acromio claviculaire. Le diagnostic est clinique avec une déformation de l’épaule avec un coup de hache externe, signe de l’épaulette, avec une tête humérale qui comble le sillon delto pectoral, enfin une ascension de l'extrémité distale de la clavicule par rapport à l'acromion. La confirmation diagnostic est radiologique avec une radiographie de l’épaule de face et de profil qui permet une analyse des deux luxations et de les classer selon leur classification respective, elle a aussi pour intérêt la recherche de fracture associée. La TDM n'est pas systématique en urgence, elle pourra mettre en évidence des lésions chondrales associée. Il s'agit d'une urgence thérapeutique, la réduction de la luxation de l’épaule doit être réalisé en premier sous anesthésie générale. L'attitude par rapport à la luxation acromio claviculaire dépend de son stade, chez notre patient l'indication chirurgicale a été posée en urgence différée.

**Figure 1:**
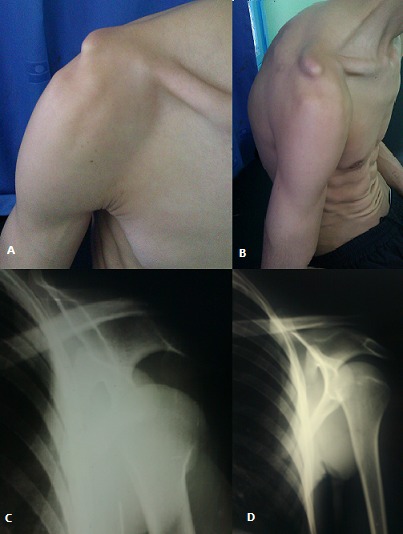
A) aspect clinique de la disjonction acromio claviculaire de face; B) aspect clinique de la disjonction acromio claviculaire de profil; D)radiographie de l'épaule de face avant réduction montrant l'association luxation gléno humérale et disjonction acromio claviculaire; E) radiographie post réduction de la luxation de l'épaule montrant la luxation acromio claviculaire

